# Occurrence of polybrominated diphenyl ethers and benzotriazole UV stabilizers in the hadal amphipod *Hirondellea gigas*

**DOI:** 10.1016/j.isci.2023.107054

**Published:** 2023-06-07

**Authors:** Ryota Nakajima, Tetsuro Ikuta, Kazumasa Oguri, Heather Ritchie

**Affiliations:** 1 Research Institution for Global Change, Japan Agency for Marine-Earth Science and Technology (JAMSTEC), Yokosuka, Kanagawa 237-0061, Japan; 2 HADAL and Nordcee, Department of Biology, University of Southern Denmark, 5230 Odense, Denmark; 3 RZSS WildGenes, Royal Zoological Society of Scotland, Edinburgh EH12 6TS, UK

**Keywords:** Marine organism, Marine processes, Environmental science

## Abstract

The accumulation of polybrominated diphenyl ethers (PBDEs) and benzotriazole UV stabilizers (BZT-UVs) were examined in the hadal amphipod *Hirondellea gigas* caught from a near-land trench off the Japan island (9200 m). *H. gigas* were collected from two distinct sites: one is located at the outlet of submarine canyons directly connected to land and the other is apart from the outlet and geographically isolated from the first site. The level of the PBDEs in *H. gigas* at the canyon outlet (mean 219 ng/g lipid weight (l.w.)) was significantly higher than that in the isolated site (mean 42 ng/g l.w.) and BZT-UVs were only detected within the first site (mean 1.5 ng/g wet weight). In addition to vertical transport from the surface water, near-land trenches associated with submarine canyons and troughs may have more complex influx of contaminants through horizontal transportation from the land, resulting in more severe contamination.

## Highlights

• PBDEs and BZT-UVs were examined in amphipods in the hadal zone (9200 m)
• PBDEs burden was greater in the amphipods near-land trench with canyon/trough
• BZT-UVs such as UV-328 can be long-range transported to hadal depths

## Graphical abstract

**Figure:**
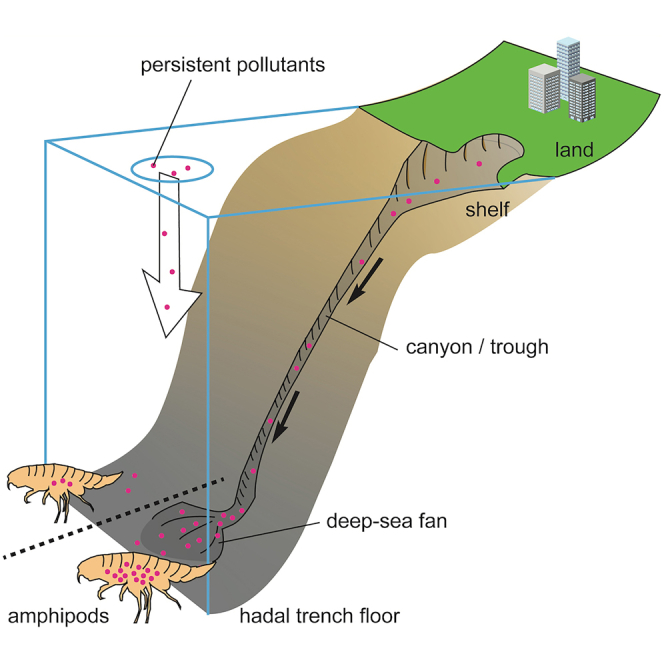

## Introduction

Over the last few decades, deep-sea ecosystems have been under an increasing threat from numerous anthropogenic impacts including contamination by persistent organic pollutants (POPs). Due to their hydrophobic properties, POPs are easily incorporated into particulate organic matter (POM) where they are transported vertically from the surface water accumulating in the deep-sea environment.^1^ As a result, POPs such as polychlorinated biphenyls (PCBs) and polybrominated diphenyl ethers (PBDEs) have been ubiquitously detected in a range of deep-sea animals (PCBs 1–12,400 ng/g lipid weight and PBDEs 0.1–517 ng/g lipid weight)^2^^,^^3^^,^^4^^,^^5^^,^^6^^,^^7^^,^^8^^,^^9^^,^^10^^,^^11^^,^^12^ and deep-sea sediments (PCBs <0.001–4 ng/g dry weight and PBDEs 0.05–0.3 ng/g dry weight).^11^^,^^13^^,^^14^^,^^15^ Several studies have shown that the levels of POPs in the deep-sea environment can be higher than those recorded in shallow waters^16^ and, as such, the deep sea is considered to be a potential sink for these persistent contaminants.^17^

At present, most research on POPs contamination in the deep sea is limited to the continental shelf (<2000 m) with only a few studies investigating abyssal (3500–6000 m) and hadal depths (6000–11000 m).^18^ This is largely due to sampling difficulties, inaccessibility, and the high cost of sampling associated with abyssal and hadal research. Investigations at these depths are essential to furthering our knowledge of POPs contamination and to allow for the monitoring of contamination in the deep sea. This is especially important for hadal trenches which may be an ultimate sink for anthropogenic pollutants due to their typical V-shaped cross section that concentrates these pollutants along the axis toward the deeper points.^19^^,^^20^

Recently, high concentrations of PCBs and PBDEs have been found in lysianassoid amphipods from the deepest, remote ocean trenches (>10000 m) such as the Mariana and the Kermadec trenches.^18^^,^^21^^,^^22^ These findings provide insights into the bioaccumulation and long-range transportation of harmful substances to full ocean depth. Although these studies provide valuable information on the levels of POPs at hadal depths in remote trenches, studies from trenches close to land with urbanized and densely populated area are lacking. Such near-land trenches might experience higher levels of POPs and/or other anthropogenic contaminants through transportation of water-borne particles via canyons and troughs connected to the land.^23^

**Figure 1:**
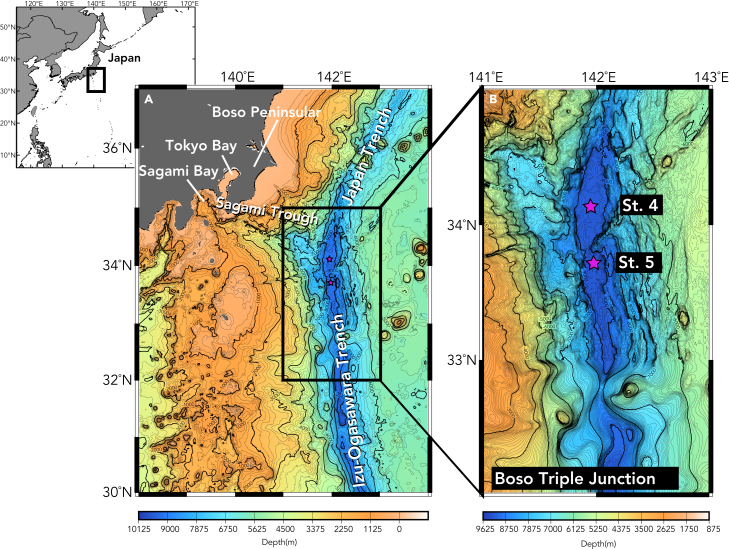
Map of the study area (A) The Boso Triple Junction off the east coast of Japan. (B) Seafloor map of the sampling sites. St. 4 (9218 m) and St. 5 (9232 m). The seabed at St. 4 is connected to Sagami Bay through the straight V-shaped slope of the Sagami Trough. Color denotes depth (m).

The Boso Plate Triple Junction is a trench-trench-trench triple junction off the Boso Peninsular on the east coast of Japan, with a hadal depth of over 9000 m.^24^ It is the meeting point of the Japan Trench to the north, the Izu-Ogasawara Trench to the south, and the Sagami Trough to the west (Figures 1A and 1B). The triple junction is some 200 km away from land but land-based particles can be transported to the triple junction seabed through submarine canyons on the slope of the Sagami Trough which act as a depocenter for particles from the Japan islands.^25^ As such, the hadal environment of the triple junction may be an area severely polluted by anthropogenic contaminants, including POPs, and the level of contaminants could be higher in near-land trenches compared to remote trenches.

Here, we examined the accumulation of PBDEs and benzotriazole UV stabilizers (BZT-UVs), one of the emerging persistent contaminants extensively used for plastic additives, in the lysianassoid amphipod *Hirondellea gigas* caught from the Boso Triple Junction (9200 m). As lysianassoid amphipods are one of the most significant members of trench ecosystems around the globe, they provide a unique opportunity for investigating pollutants in deep-sea ecosystems. We test the hypothesis that the levels of POPs from the hadal amphipods in the Triple Junction are higher in the area close to the outlet of submarine canyons than in the area isolated from the outlet. Moreover, given the feeding ecology and known plastic ingestion,^26^ we expect that the hadal amphipods may also have been exposed to plastic additives such as BZT-UVs. One of the BZT-UVs (UV-328) has recently been included in the list of Annex A of the Stockholm convention.^27^ Since little is known about the long-range transport potential of BZT-UVs, it is important to investigate these persistent pollutants at hadal depths. If BZT-UVs are detected in these hadal amphipods, this would provide additional evidence of the long-range transport of these contaminants.

## Results

**Figure 2:**
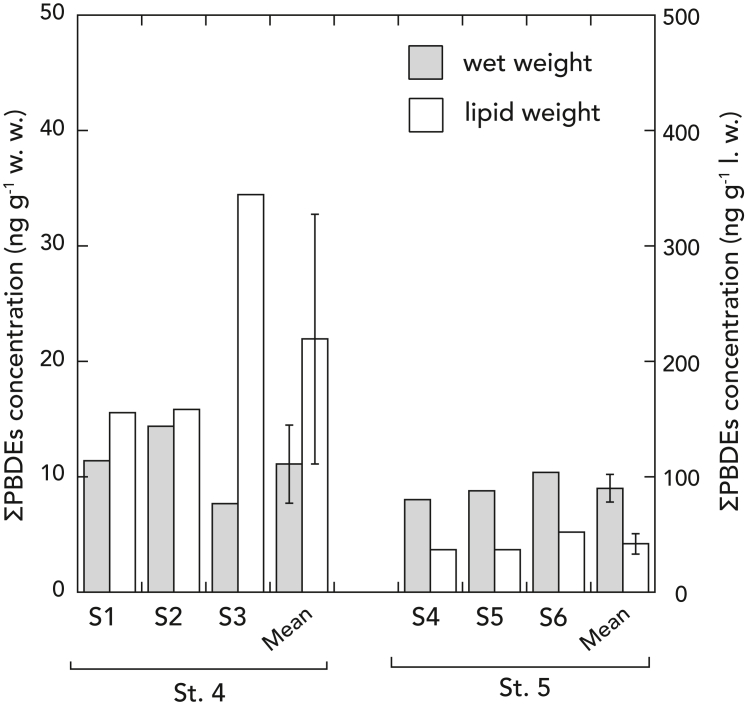
The concentration of PBDEs in *Hirondellea gigas* from the Boso Triple Junction, Japan The concentration (ng g^−1^) in wet weight (w.w.) and lipid weight (l.w.) of PBDEs at St. 4 and 5. S denotes specimen. Error bars in mean indicate standard deviation.

PBDEs were detected in all specimens of the hadal amphipod *H. gigas* collected at the site close to the outlet of submarine canyons on the slope of Sagami Trough (St. 4 the Mogi submarine fun) and the site apart from the outlet (St. 5) in the Boso Triple Junction (depth: 9218–9232 m). The mean concentration of PBDEs per lipid weight (l.w.) at St. 4 (mean ± SD: 219.4 ± 108.1 ng/g l.w.) was one order of magnitude higher than that found at St. 5 (41.9 ± 8.9 ng/g l.w.) with a significant difference (*t*-test, p = 0.047) (Figure 2). PBDE concentration per wet weight (w.w.) was similar in both sites (mean ± SD: 11.1 ± 3.4 ng/g w.w. at St. 4; 9.0 ± 1.2 ng/g w.w. at St. 5) due to higher lipid content in the individuals of *H. gigas* at St. 5 (Table S1).

**Figure 3:**
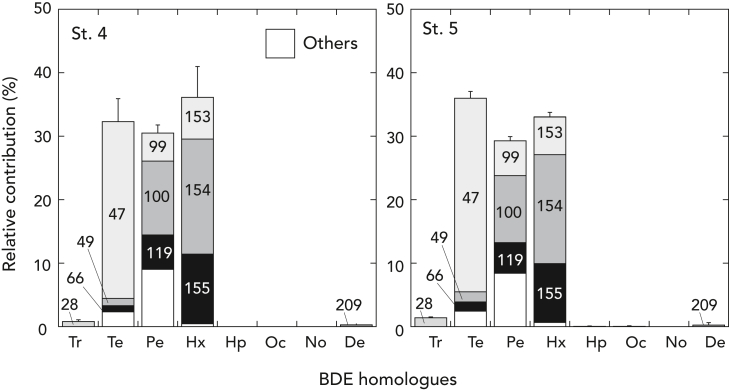
Relative contribution of BDE homologs in *Hirondellea gigas* at St. 4 and 5 Each column represents a BDE homolog as follows: Tr, tri-BDE; Te, tetra-BDE; Pe, penta-BDE; Hx, hexa-BDE; Hp, hepta-BDE; Oc, octa-BDE; No, nona-BDE; De; deca-BDE. Columns are further split by congener profiles where major congeners are listed by number and all other congeners are represented by a blank box. Error bars in mean indicate standard deviation.

The distribution patterns of BDE homologs were similar in both sites, with tetra-BDEs (32.3%–36.0%), penta-BDEs (29.3%–30.5%), and hexa-BDEs (33.1%–36.1%) providing similar contributions to the total PBDEs (Figure 3, Table S1). Tetra-BDEs were primarily composed of BDE-47 (84.7%–86.2%), followed by BDE-49 (3.6%–4.4%) and BDE-66 (3.0%–4.0%). Penta-BDEs were mainly comprised of BDE-100 (36.1%–38.2%), followed by BDE-99 (14.5%–18.6%) and BDE119 (16.6%–17.8%). The three major congeners in hexa-BDEs were BDE-154 (50.2%–51.8%), BDE-155 (28.0%–30.4%), and BDE-153 (18.0%–18.2%). Tri-BDE accounted for 0.8%–1.4% of the total PBDEs and was primarily composed of BDE-28. Hepta-, octa-, and nona-BDE were consistently not detected or below detection limit in all samples. Although very minor (<0.3%) and under quantification limit, deca-BDE (BDE-209) was also detected in *H. gigas* at both sites.

**Figure 4:**
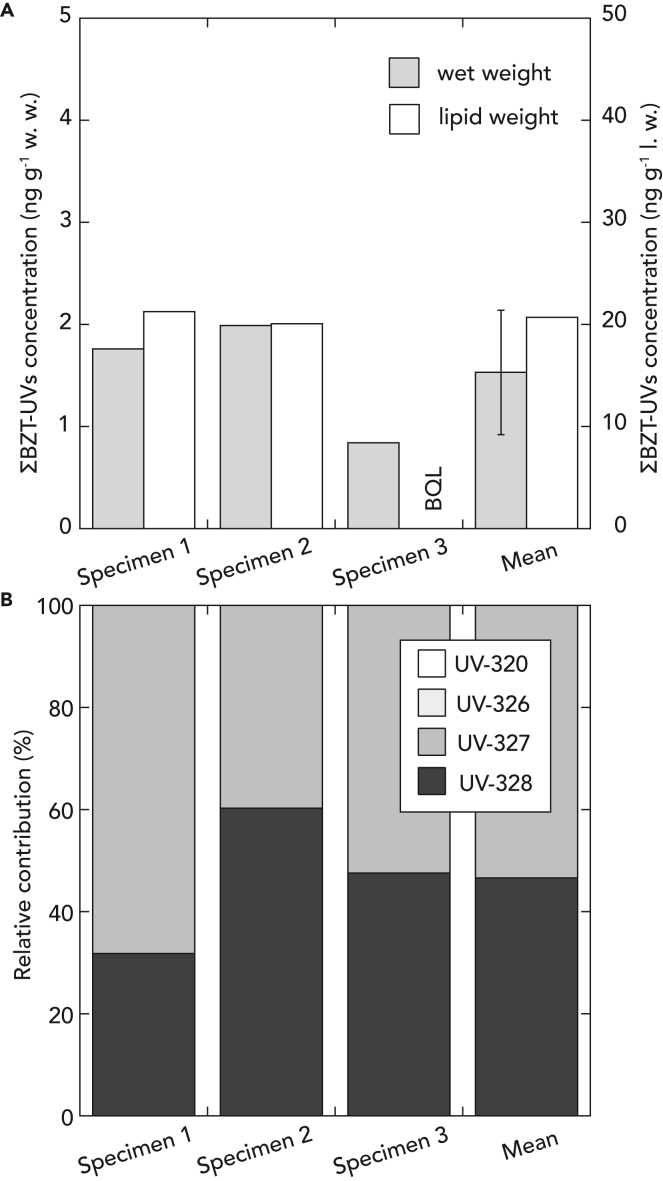
The concentration of benzotriazole UV stabilizers (BZT-UVs) in *Hirondellea gigas* from the Boso Triple Junction (St. 4), Japan (A) The concentrations (ng g^−1^) in wet weight (w.w.) and lipid weight (l.w.) of BZT-UVs. Error bars in mean indicate standard deviation. BQL: below quantitative limit. (B) Relative contribution (%) of four BZT-UVs (UV-320, 326, −327, and −328) in each specimen.

BZT-UVs were detected only in *H. gigas* collected at St. 4, ranging from 0.84 to 1.99 ng/g wet weight (w.w.) (mean ± SD: 1.53 ± 0.61 ng/g w.w.). BZT-UVs ranged from 20.1 to 21.3 ng/g l.w. with one specimen below the quantitative limit due to low lipid content (2.4%) (Figure 4A). BZT-UVs were primarily composed of UV-327 (39.7%–68.2% in w.w.) and UV-328 (31.8%–60.3% in w.w.), where UV-320 and UV-326 were consistently not detected (Figure 4B).

## Discussion

Deep-sea trench systems were once considered a pristine environment free from anthropogenic impacts due to their extremely deep waters and their remote locations. However, PBDEs were detected in all specimens, and BZT-UVs in some specimens, of the hadal amphipod *H. gigas* collected at the Boso Triple Junction. This highlights the widespread contamination of POPs and other persistent contaminants in the hadal zone.^20^^,^^28^

The level of PBDEs found in *H. gigas* at the site close to the Mogi submarine fan, the outlet of Sagami Trough (St. 4, mean 219 ng/g l.w.), was one order of magnitude higher than the levels found at the site away from the outlet (St. 5, mean 42 ng/g L.w.). BTZ-UVs were only detected from the individuals of *H. gigas* at St. 4 (mean 1.5 ng/g wet weight). These discrepancies in the concentrations of PBDEs and BZT-UVs between the two sites in the Boso Triple Junction are somewhat unclear, but differences in the transportation process of contaminants may be responsible. Studies have shown that hadal amphipods preferentially utilize labile and fresh POM and carcasses derived from the surface water.^29^ As PBDEs released into the marine environment are incorporated into POM and carcasses due to their hydrophobic properties,^30^ sinking POM and carcasses are likely to be important carriers of PBDEs into the study sites.^22^

In addition to this vertical transportation, there may be horizontal transportation of contaminants to St. 4 with water-borne particles. As shown in Figure 1B, St. 4 on the Triple Junction is directly connected to Sagami Bay off the Japan mainland through the straight V-shaped downward slope of the Sagami Trough. Previous studies showed that sediments off the Boso Peninsula are transported along the Sagami Trough and ultimately into the Boso Triple Junction, especially during earthquake events causing submarine landslides.^31^^,^^32^ This downward seafloor transportation of sediments may also enable the transportation of anthropogenic contaminants, such as PBDEs, together with POM and possibly polluted carrion and microplastics^33^ from Sagami Bay to the seabed (9200 m, St. 4) of the Triple Junction.^24^^,^^25^ Sagami Bay and the neighboring Tokyo Bay are associated with urban cities in a densely populated region including Tokyo metropolitan city. Many submarine channels, including the Tokyo Canyon from Tokyo Bay, drain into the Sagami Trough as tributary channels,^25^ and thus Sagami Trough would be one of the significant pathways of anthropogenic pollutants from the populated regions in the Japan islands. In contrast, there is unlikely to be a significant supply of water-borne particles to St. 5 through the Sagami Trough due to the distance to the outlet (end) of the channel and the only passage between Sts. 4 and 5 are narrow (Figure 1B). This difference in geological features may form a plausible explanation for the differences in the concentration of contaminants between St. 4 and St. 5 in the Boso Triple Junction.

There are three previous reports that examined the contamination level of PBDEs in hadal amphipod *H. gigas* from remote ocean trenches including the Mariana Trench (6025–10,911 m).^18^^,^^21^^,^^22^ Direct comparison is not possible with these studies due to the different numbers of congeners being examined: Jamieson et al. (2017)^18^ investigated seven congeners, Cui et al. (2020)^21^ measured nine congeners, and Xie et al. (2023)^22^ measured 16 congeners (Table 1). Still if the comparisons are to be made by adjusting the number of congeners, it would be as follows. The PBDEs levels of *H. gigas* at St. 4 in the Boso Triple Junction (mean 154 ng/g l.w. with seven congeners, 157 ng/g l.w. with nine congeners, and 162 ng/g l.w. with 16 congeners) were one order of magnitude higher than the previously reported levels in the Mariana Trench (Table 1). In contrast, the PBDE concentrations found in *H. gigas* at St. 5 (mean 30–31 ng/g l.w. with seven/nine/16 congeners) are in the same range as those reported in the Mariana Trench. These comparisons suggest that St. 5 in the Boso Triple Junction, where the direct supply of contaminants from land is less likely, may solely rely on the vertical transportation of contaminants from the surface water, similar to remote ocean trenches such as the Mariana Trench. On the contrary, higher PBDEs concentration in *H. gigas* found at St. 4 supports the hypothesis that the contaminant levels in a near-land trench can be higher than those in ocean trenches if there are canyons/trenches that are directly linked to land. This highlights the need for further studies to investigate persistent pollutants in amphipods in other near-land trenches such as the Atacama Trench,^15^^,^^20^ which is an important step into elucidating possible transportation of contaminants in the hadal zone.

**Table 1:** Summary of the concentrations of PBDEs (ng g^−1^ lipid weight) in *Hirondellea gigas* from the Mariana Trench and the Boso Triple Junction

Trench	Depth (m)	Examined congeners	ΣPBDEs (ng/g lipid weight)	Source
Mariana	7841–10250	28, 47, 99, 100, 153, 154, 183	10–60 (25)	Jamieson et al.^18^
Mariana	7125–10911	28, 47, 66, 99, 100, 138, 153, 154, 183	6–22 (10)	Cui et al.^21^
Mariana	6025–10853	17, 28, 47, 66, 71, 85, 99, 100, 138, 153, 154, 183, 191, 206, 207, 209	6–69 (20)	Xie et al.^22^
Boso Triple Junction (St. 4)	9218	28, 47, 99, 100, 153, 154, 183	105–250 (154)	This study
		28, 47, 66, 99, 100, 138, 153, 154, 183	107–256 (157)	This study
		17, 28, 47, 66, 71, 85, 99, 100, 138, 153, 154, 183, 191, 206, 207, 209	110–262 (162)	This study
Boso Triple Junction (St. 5)	9232	28, 47, 99, 100, 153, 154, 183	26–36 (30)	This study
		28, 47, 66, 99, 100, 138, 153, 154, 183	27–37 (30)	This study
		17, 28, 47, 66, 71, 85, 99, 100, 138, 153, 154, 183, 191, 206, 207, 209	28–38 (31)	This study

The values in parentheses indicate the means of the data presented within the studies.

Our results show that the hadal amphipods exhibited a high contribution of BDE-47, comparable to those reported in earlier studies from the Kermadec and Mariana trenches.^18^^,^^21^ The high contribution of BDE-47 may be attributed to the high bioaccumulation potential of these congeners as well as the results of debromination of higher congeners in biota tissues.^34^ The high contribution of BDE-47 is typical for amphipods due to higher biomagnifications of BDE-47 within the aquatic food web.^35^ BDE-47 has also been proven to be formed via debromination of higher congeners during the metabolism.^35^ The ratios of BDE-47/(BDE-47+BDE-99) with a mean ratio of 0.85–0.86 in *H. gigas* in this study were larger than the ratio of the corresponding technical penta-BDE mixture (0.4–0.5), indicating degradation of less abundant brominated BDEs (such as BDE-99).^36^ The other predominant congeners were BDE-100, 119, 153, 154, and 155. Similar BDE profiles were reported from deep-sea predatory fishes in Japan.^4^^,^^37^ Specifically, BDE-154 and BDE-155 represented an important proportion of hexa-BDE in *H. gigas* in this study, which is in accordance with previous findings on deep-sea sharks in Suruga Bay, near Sagami Bay, Japan.^4^ These congeners are probably products of the debromination of higher brominated congeners such as BDE-183 and BDE-209, the main congeners in the technical octa-BDE and deca-BDE mixtures, respectively.^38^ The higher brominated congeners of octa- and nona-BDEs were consistently not detected or below the detection limit in *H. gigas*, suggesting the low bioaccumulation potential and/or the low bioavailability of these congeners.^39^ Similarly, the low detection rate of BDE-209 in *H. gigas* was also likely due to lower bioavailability of these congeners, though deca-BDE commercial mixtures have been largely consumed in countries around the Asia-Pacific Ocean.^40^ However, Xie et al. recently showed a strong contribution of BDE-209 in the tissue of hadal amphipods.^22^ Further investigations are proposed in order to obtain a full understanding of PBDEs and other POPs contaminations in hadal amphipods including their temporal variation.^41^

BZT-UVs have been extensively used as additives in many types of plastic products and personal care products to reduce UV-induced damage/degradation and color failure/yellowing.^42^^,^^43^ BZT-UVs have been ubiquitously found in various media,^44^^,^^45^ and the deep-sea environment is not an exception as UV-327 and UV-328 were detected in the hadal amphipods at St. 4 in this study. The occurrence of BZT-UVs in biota tissue is used as one of the indicators for plastic ingestion.^46^ Given the seafloor transportation of sediments and contaminants from Japan island to the seabed at St. 4 through the V-shaped slide of Sagami Trough, it is not surprising that plastic debris (microplastics) containing BZT-UVs are being directly transported into the seabed at St. 4. Indeed, a previous study has shown that massive amounts of microplastics are being transported from Sagami Bay to the open ocean during extreme weather events such as tropical cyclones.^47^ In the Mariana Trench, the hadal amphipod *H. gigas* has been shown to ingest microplastics^26^ and, as such, the occurrence of BZT-UVs in *H. gigas* in this study may be attributed to plastic ingestion and/or ingestion of carcasses contaminated with plastic additives.

It is difficult to directly compare the BZT-UVs concentrations in *H. gigas* from this study (St. 4) with other organisms from different locations because bioaccumulation depends on multiple factors such as local anthropogenic contamination and feeding strategies, habitat, longevity, or species-specific physiology as already reported for other contaminants in several animals.^48^ Notwithstanding, the BZT-UVs levels of *H. gigas* in this study (mean 1.53 ng/g w.w.) are in the same range as those reported in tidal and shallow water arthropods and mollusks from the Ariake Sea, Japan^49^ and coastal fishes from Spain.^50^ Despite the deep sea being a potential sink for persistent contaminants,^17^ BZT-UVs may still be a relatively new pollutant in hadal environments, which could explain the lack of BZT-UVs contamination in *H. gigas* at St. 5.

Since BZT-UVs are recognized as an emerging contaminant due to their high persistency, bioaccumulation, and toxic properties, it is important to understand the extent and long-range transport of these pollutants. Previous studies have confirmed the contaminations of ringed seals from Canadian Arctic sites (UV-326, -329, and −350)^51^ and in seabirds from several remote islands in the Pacific, Atlantic, and Indian Oceans (UV-P, −326, −329, −328, −327, and −234).^46^ Here, we have shown evidence of long-range transport of UV-327 and UV-328 to the hadal zone (9200 m) in the deep-sea environment.

In summary, the transport of persistent contaminants to hadal trenches may be more complex than previously thought in near-land trenches as contaminants can be transported from the mainland to a near-land trench system through horizontal transportation via canyons and troughs. We also confirm the hypothesis that the contamination of plastic additives such as BZT-UVs affects even organisms in the hadal zone which is evidence of long-range transportation of BZT-UVs.

### Limitations of the study

It should be noted that the number of samples used in this study was small due to the sampling difficulties in such an extremely deep environment (over 9000 m water depth) and more data are needed to understand the true extent of POPs at hadal depths. Yet this study provides important baseline data for PBDEs and other contaminants in hadal amphipods especially as we have recorded PBDE concentrations higher than any previously published in hadal animals as shown previously.

## STAR★Methods

### Key resources table

**Table:** 

REAGENT or RESOURCE	SOURCE	IDENTIFIER
**Biological samples**		

Amphipods (*Hirondellea gigas*)	Boso Triple Junction, Japan	N/A

**Chemicals, peptides, and recombinant proteins**		

Native PBDE Solution/Mixture, BDE-MXE	Wellington Laboratories	Cat#49847-77
Mass-Labelled PBDE Solution/Mixture, MBDE-MXG-STK	Wellington Laboratories	Cat#49880-04
Mass-Labelled PBDE Internal Standard Solution, MBDE-ISS-G	Wellington Laboratories	Cat# 49880-05
2-(3,5-Di-tert-butyl-2-hydroxyphenyl)2H-benzotriazole, UV-320	AccuStandard	PLAS-UV-010N; CAS: 3846-71-7
2-tert-butyl-6-(5-chloro-2H-benzotriazol-2-yl)-4-methylphenol, UV-326	AccuStandard	PLAS-UV-009N; CAS: 3896-11-5
2,4-Di-tert-butyl-6-(5-chloro-2H-benzotriazol-2-yl)phenol, UV-327	AccuStandard	PLAS-UV-011N; CAS: 3864-99-1
2-(2H-Benzotriazol-2-yl)-4,6-di-tert-pentylphenol, UV-328	AccuStandard	PLAS-UV-012N; CAS: 25973-55-1
Potassium hydroxide (KOH)	Fujifilm-Wako	162-21813; CAS: 1310-58-3
Ethanol for Pesticide Residue and Polychlorinated Biphenyl Analysis	Fujifilm-Wako	053-07011; CAS: 64-17-5
Hexane for Pesticide Residue and Polychlorinated Biphenyl Tests	Fujifilm-Wako	083-07911; CAS: 110-54-3
Acetone for Pesticide Residue and Polychlorinated Biphenyl Tests	Fujifilm-Wako	011-19201; CAS: 67-64-1
Sodium chloride (NaCl) for Pesticide Residue and Polychlorinated Biphenyl Tests	Kanto Kagaku	37144-1B; CAS: 7647-14-5
Sodium sulfate (Na_2_SO_4_) for Pesticide Residue and Polychlorinated Biphenyl Tests	Kanto Kagaku	6639-1B; CAS: 7757-82-6
Acetonitrile (CH_3_CN) for Pesticide Residue and Polychlorinated Biphenyl Analysis	Fujifilm-Wako	013-19401; CAS: 75-05-8
Silica gel 60 for column chromatography	Kanto Chemical	7748-1M; CAS: 7631-86-9
Active carbon-dispersed silica gel reversible column	Kanto Chemical	Cat# 01894-96

**Deposited data**		

Raw and analyzed data	This paper	Tables S1 and S2

**Software and algorithms**		

KaleidaGraph	Synergy Software	https://www.synergy.com
R	R Core Team	https://www.R-project.org

**Other**		

NH_2_ cartridge, Bond Elut Jr-NH_2_	Agilent Technologies	https://www.agilent.com/en/product/sample-preparation/solid-phase-extraction-spe/bond-elut-nh2
Rotary evaporator, N-1300V-W	EYELA	https://eyelaworld.com/products/products_tax/ev/ev02/
GC, 7890 series	Agilent Technologies	https://www.agilent.com/en/product/gas-chromatography/gc-systems/7890b-gc-system
HRMS, AutoSpec-Ultima	Waters	https://www.waters.com/nextgen/jp/ja/library/application-notes/2003/parallel-dual-column-gc-ms-with-the-autospec-ultima-nt.html
BPX-DXN column	Kanto Chemicals	https://cica-web.kanto.co.jp/CicaWeb/servlet/wsj.front.LogonSvlt?lang=En&ReqItem=95106-05
BP1 column	SGE Analytical Science	https://www.analytics-shop.com/us/sg054090
ACQUITY UPLC	Waters	https://www.waters.com/waters/en_US/ACQUITY-UPLC-Detectors/nav.htm?cid=514217&locale=en_US
6500 QTRAP tandem mass spectrometer	Sciex	https://sciex.com/products/mass-spectrometers/qtrap-systems/qtrap-6500-system
ACQUITY UPLC BEH C18 column	Waters	https://www.waters.com/nextgen/us/en/shop/columns/186002350-acquity-uplc-beh-c18-column-130a-17--m-21-mm-x-50-mm-1-pk.html
Free-Fall Camera Lander system, FFC11K	Maeda et al.^52^	https://lsc-pagepro.mydigitalpublication.com/publication/?i=686620&p=22&view=issueViewer

### Resource availability

#### Lead contact

Further information and requests for resources and reagents should be directed to and will be fulfilled by the lead contact, Ryota Nakajima (nakajimar@jamstec.go.jp).

#### Materials availability

This study did not generate new unique reagents. All key recourses are listed in the key resources table. Further information and requests for resources and reagents should be directed to the lead contact.

### Experimental model and subject details

All investigations were performed on amphipods, *Hirondellea gigas*. All amphipods were collected by bait traps from Boso Triple Junction, off the Japan Island. The animal is not endangered or protected species.

### Method details

#### Sample collection

The drainage system in the region off the Boso Peninsula transports clastic sediments from Sagami Bay along the downward slope of Sagami Trough (Figure 1A), through the Sagami and Boso submarine canyons, and ultimately into the Boso Triple Junction, forming the Mogi submarine fan.^31^ *Hirondellea gigas* individuals were collected at two sites in the seabed of the Boso Triple Junction: St. 4 with a 9218 m depth (34°20.4296′N; 142°00.6006′E) and St. 5 with a 9232 m depth (33°46.4374′N; 141°56.1556′E) in September 2019 (Figures 1A and 1B). St. 4 is located in the area of Mogi submarine fan, i.e. the outlet (end) of the Sagami Trough which is directly connected to Sagami Bay. In contrast, St. 5 is apart from the outlet and the only passage between Sts. 4 and 5 is narrow (Figure 1B).

The location for this study was within the exclusive economic zone (EEZ) of Japan and not protected in any way. No specific permits were required for the described field studies and sample collection. The study site did not involve any endangered or protected species.

The samples were collected using a bait trap attached to a Free-Fall Camera Lander system (FFC11K)^52^ on September 4 at St. 4 and September 11 at St. 5, on R/V Yokosuka cruise YK19-11. A cut of mackerel (Scombridae) was used as bait. Specimens were taken in triplicate with each repeat containing ∼10 individuals, as well as a portion of the bait. These were placed in pre-combusted (500°C, 4 h) glass jars with combusted foil placed over the top and stored in a deep-freezer at −20°C for subsequent analyses of PBDEs, BZT-UVs and lipids.

#### Analyses of PBDEs and BZT-UVs

##### Chemical and materials

The standards for the 27 PBDE congeners (BDE-3, 7, 15, 17, 28, 47, 49, 66, 71, 77, 85, 99, 100, 119, 126, 138, 153, 154, 156, 183, 184, 191, 196, 197, 206, 207, 209) (BDE-MXE) were obtained from Wellington Laboratories Inc. (Guelph, ON, Canada). ^13^C_12_-labeled PBDEs (isomer #3, 15, 28, 47, 99, 100, 126, 153, 154, 183, 197, 207 and −209) (MBDE-MXG-STK) spiked as internal standards and ^13^C_12_-labeled BDE-79, -138, and −206) (MBDE-ISS-G) as syringe spikes were obtained from Wellington Laboratories Inc. (Guelph, ON, Canada). Analytical standards of four BZT-UVs (UV-320, UV-326, UV-327, and UV-328) were purchased from AccuStandard, Inc. (New Haven, CT, USA) (See key resources table for detail).

Pesticide residue analytical-grade solvents such as hexane, acetone, and sodium sulfate were purchased from Fujifilm-Wako Pure Chemical (Osaka, Japan) and Kanto Chemical (Tokyo, Japan). Silica gel and active carbon-dispersed silica gel were obtained from Kanto Chemical (Tokyo, Japan) (See key resources table for detail).

##### Sample extraction and cleanup

All the sample extraction, cleanup and instrumental analyses of PBDEs, BZT-UVs and lipids were conducted in the laboratory of IDEA Consultants (Tokyo, Japan).

The PBDEs extraction procedure used in this study is similar to those described in previous reports.^53^^,^^54^^,^^55^^,^^56^ Homogenized sample (4 g wet) was collected in a 50 ml centrifuge tube and spiked with surrogates containing 5 ng of ^13^C_12_-labeled PBDEs as internal standards. Samples were extracted twice with a mixture of acetone/hexane (1:2 *v*/*v*; 20 mL) in a homogenizer (IKA Werke, Germany) for 2 min. After centrifugation (2500 rpm, 5 min), the organic phase was washed with a water solution of 5% NaCl, and then dehydrated with anhydrous sodium sulfate. The extracted samples were treated with concentrated sulfuric acid (H_2_SO_4_) and then concentrated to 10 mL in a rotary evaporator (N-1300V, EYELA, Japan). The extracted samples were then partitioned twice using a mixture of acetonitrile/hexane (1:1 v/v; 50 mL). The hexane extract was concentrated to 1 mL by the rotary evaporator. The extract was passed through a multi-layer silica gel column that consisted of 1 g silica gel, 4 g acidic silica gel, and 2 g anhydrous sodium sulphate and eluted with 100 mL of hexane. The cleaned sample extracts were concentrated to 1 mL and subsequently fractionated by the active carbon column packed with 1 g activated carbon-dispersed silica gel (Kanto Chemical, Tokyo, Japan). The sample extract was loaded onto the carbon column and washed with 25 mL hexane, and this first fraction was discarded. The second fraction was then eluted with 40 mL of 25% dichloromethane/hexane. The eluent was concentrated and spiked with injection internal standards (^13^C_12_-labeled BDE-79, -138, and −206) as syringe spikes prior to gas chromatography-high resolution mass spectrometry (GC-HRMS) analysis.

BZT-UVs were analyzed using a method described previously within Watanabe and Noma (2010).^57^ Homogenate samples (2 g, wet) were refluxed with 10 ml of 0.6 mol/l KOH-Ethanol solution at 90°C for 2 hours. The target compounds in the ethanol extract were transferred to 80 ml of hexane in a separatory funnel and extracted twice with liquid-liquid extraction. The resulting extracts were dehydrated with anhydrous sodium sulfate. The hexane extracts were concentrated to 2 mL using a rotary evaporator (N-1300V-W, EYELA, Japan). The solution was passing through NH_2_ cartridge (500 mg, Bond Elut Jr-NH_2_, Agilent Technologies, CA, USA), which were pre-washed by 10 mL of hexane, and the target compounds were eluted by 4 mL of hexane. Hexane which passed through the NH_2_ cartridge was dried under a gentle N_2_ stream. Two mL of methanol was added to the dried substance and again concentrated to 1 mL using a gentle N_2_ stream for subsequent instrumental analysis. Total lipid contents of the samples were determined using a single-step extraction method following the conventional chloroform-methanol extraction method.^58^

##### Instrumental analysis

Measurements of PBDEs were performed by a GC (7890 series, Agilent) combined HRMS (AutoSpec-Ultima, Waters, MA, USA) with a BPX-DXN column (30 m length ×0.25 mm i.d., Kanto Chemical, Tokyo, Japan) for separating mono- to hepta-BDEs, and a BP1 column (15 m length × 0.25 mm i.d., SGE Analytical Science, Australia) for separating octa- to deca-BDEs. The inlet temperature was 260°C for the measurement of mono- to hepta-BDEs and 250°C for the measurement of octa- to deca-BDEs. The interface of GC to MS was set at 290°C. One L solution was injected by splitless mode and He carrier gas was used at 1 mL min^−1^. The program of GC oven for the measurement of mono- to hepta-BDEs was as follows: initial temperature of 130°C (held for 1 min) was raised to 180°C at 20°C min^−1^, from 180 to 280°C at 5°C min^−1^, from 280 to 330°C at 3°C min^−1^ and then held for 5 min. The program for the measurements of octa-to deca-BDEs was as follows: initial temperature of 180°C (held for 2 min) was raised to 310°C at 5°C min^−1^ and then held for 6 min. Electron impact and selected ion monitoring mode (EI-SIM) was used for the GC/MS analyses.

Chromatographic separation for BZT-UVs was performed on an ultra-performance liquid chromatography tandem mass spectrometer (UPLC-MS/MS) system. The UPLC-MS/MS consisted of a ACQUITY UPLC (Waters) and a 6500 QTRAP tandem mass spectrometer (Sciex, MA, USA). The UPLC column was a ACQUITY UPLC BEH C18 column (50 × 2.1 mm, 1.7 μm particle size) (Waters). The column temperature was set at 40°C. 0.5 mM ammonium acetate (A) and methanol (B) were used as mobile phases. Gradient elution was performed with an initial mobile phase of 60:40 (A:B, v/v), increasing to 5:95 over 5 min, from 5:95 to 1:99 over 10 min, and held constant for 5 min before returning to the initial mobile phase composition. The flow rate was set at 0.2 ml min^−1^. The samples were introduced into the mass spectrometer with an injection volume of 15 μl. The mass spectrometer was operated in APCI -negative SRM mode.

#### Quality assurance/quality control (QA/QC)

The sample treatment process was performed in a clean cabinet to avoid background contamination. All glass and stainless-steel equipment and utensils were cleaned using demineralized water and neutral detergent, dried at high temperature (400°C), then rinsed with acetone and hexane before use and between samples. To ensure the quality of PBDEs and BZT-UVs measurements, the linearity of the method was checked using calibration curves made from standard solutions at five concentration levels (1–200 pg injected in triplicate), with coefficients of determination >0.98 for each compound. The recoveries (*n* = 6) of PBDEs and BZT-UVs from spiked samples ranged from 89 to 163% and 83–99%, and relative standard deviations (RSDs) were below 14% for PBDE and 26% for BZT-UVs, respectively (Table S2). Both the spike and blank methods were processed for each batch to monitor background interference during the whole sample preparation and analytical process. Procedural blanks were run at a rate of one in every ten samples and did not affect the sample concentrations.

The method detection limits (MDLs) were defined as 2 times the standard deviation (SD) of the procedural blank: *MDLs* = *t* (*n* – 1, 0.05) ∗ 2 ∗ *s*, where *t*(*n*-1, 0.05) represents the Student’s *t*-value at an α level of 0.05 with *n-1* degrees of freedom, and *s* represents the standard deviation of the blank measurements in several replicates (*n* = 7 for PBDEs and *n* = 8 for BZT-UVs). The method quantification limits (MQLs) were calculated as 10 times the SD of procedural blanks. The MDLs and MQLs for PBDEs ranged from 3.3 to 20.0 pg/g w.w. and 8.5 to 60.0 pg/g w.w., respectively. The MDLs and MQLs for BZT-UVs ranged from 0.034 to 0.15 ng/g w.w. and 0.089 to 0.40 ng/g w.w., respectively (Table S2). Concentrations of PBDEs were corrected from the recoveries of surrogates. The recoveries of surrogate standards were from 32% to 100% in all analyzed samples.

Since the whole body of *H. gigas* were used for measurements of PBDEs and BZT-UVs, the concentrations of PBDEs and BZT-UVs in *H. gigas* were determined by subtracting the concentrations in the bait from those measured in *H. gigas*. The concentration of PBDE in the bait (1.4 ng/g lipid weight) accounted for 0.4–3.9% of the concentrations of the measured samples of *H. gigas*. No BZT-UVs were detected in the bait.

#### Statistical analysis

The differences in the PBDE levels between different sites were determined using a Student’s *t* test. A difference at p < 0.05 was considered significant. Analyses were performed with the R Stats Package (version 4.3.0).

## Data Availability

• All data are available in this paper and Tables S1 and S2. • This paper does not generate original code. • Any additional information required to reanalyze the data reported in this paper is available from the lead contact upon request.
