# Supplementary material for: Occurrence of polybrominated diphenyl ethers and benzotriazole UV stabilizers in the hadal amphipod Hirondellea gigas
Source: iScience. 2023 Jun 7;26(7):107054. doi: 10.1016/j.isci.2023.107054 (PMC10391725; doi:10.1016/j.isci.2023.107054)
Supplement: Document S1. Tables S1 and S2 [file mmc1.pdf]

iScience, Volume *26*

Supplemental information

Occurrence of polybrominated diphenyl ethers and benzotriazole UV stabilizers in the hadal amphipod *Hirondellea gigas*

Ryota Nakajima, Tetsuro Ikuta, Kazumasa Oguri, and Heather Ritchie

Table S1. Lipid content (%) and concentration of several major congeners and total PBDEs (ng/g lipid weight) in *Hirondellea gigas* collected from the Boso Triple Junction, Japan, Related to Figure 2

Specimen	Lipid (%)	BDE-28	BDE-47	BDE-49	BDE-66	BDE-99	BDE-100	BDE-118	BDE-119	BDE-153	BDE-154	BDE-155	ΣPBDEs
Specimen 1 St. 4	8.2	0.80	42.76	1.80	0.65	7.42	19.23	3.02	8.13	11.11	26.40	17.40	155.73
Specimen 2 St. 4	9.7	1.64	38.27	2.22	1.32	7.58	13.35	2.48	8.90	11.60	32.70	19.26	158.29
Specimen 3 St. 4	2.4	3.06	109.91	3.27	5.60	12.65	48.60	5.37	18.78	17.97	57.84	32.97	344.27
Specimen 4 St. 5	23.5	0.54	11.48	0.68	0.49	1.99	3.81	0.62	1.72	2.44	6.00	3.22	36.69
Specimen 5 St. 5	25.5	0.45	11.33	0.54	0.47	2.28	3.92	0.67	1.59	2.25	6.18	3.50	36.86
Specimen 6 St. 5	22.2	0.76	15.36	0.77	0.89	2.47	5.55	0.90	2.88	2.67	9.53	4.98	52.21

Table S2. Method detection limits (MDLs), method quantification limits (MQLs), spiked average recoveries and relative standard deviations (RSD) for PBDEs and BZT-UVs, Related to STAR Methods

Group	Compound	MDLs (pg/g ww)	MQLs (pg/g ww)	Recovery (%)	(%RSD)
PBDEs	BDE-47	8.00	8.55	163.38	5.23
	BDE-99	4.07	10.46	116.08	9.01
	BDE-153	5.75	14.79	89.25	8.29
	BDE-175	10.00	30.00	104.58	14.40
	BDE-197	9.50	24.43	92.25	13.24
	BDE-207	7.98	20.52	100.10	4.10
	BDE-209	20.00	60.00	156.84	7.95
BZT-UVs	UV-320	53.00	140.00	83.14	11.21
	UV-326	150.00	400.00	98.60	2.00
	UV-327	34.00	89.00	94.82	6.24
	UV-328	43.00	120.00	96.31	7.79
